# Attention deployment in natural scenes: Higher-order scene statistics rather than semantics modulate the N2pc component

**DOI:** 10.1167/jov.24.6.7

**Published:** 2024-06-07

**Authors:** Daniel Walper, Alexandra Bendixen, Sabine Grimm, Anna Schubö, Wolfgang Einhäuser

**Affiliations:** 1 Physics of Cognition Group, Chemnitz University of Technology, Chemnitz, Germany; 2 Cognitive Systems Lab, Chemnitz University of Technology, Chemnitz, Germany; 3 Cognitive Neuroscience of Perception & Action, Philipps University Marburg, Marburg, Germany

**Keywords:** visual search, natural scenes, visual attention, N2pc, EEG

## Abstract

Which properties of a natural scene affect visual search? We consider the alternative hypotheses that low-level statistics, higher-level statistics, semantics, or layout affect search difficulty in natural scenes. Across three experiments (*n* = 20 each), we used four different backgrounds that preserve distinct scene properties: (a) natural scenes (all experiments); (b) 1/*f* noise (pink noise, which preserves only low-level statistics and was used in Experiments 1 and 2); (c) textures that preserve low-level and higher-level statistics but not semantics or layout (Experiments 2 and 3); and (d) inverted (upside-down) scenes that preserve statistics and semantics but not layout (Experiment 2). We included “split scenes” that contained different backgrounds left and right of the midline (Experiment 1, natural/noise; Experiment 3, natural/texture). Participants searched for a Gabor patch that occurred at one of six locations (all experiments). Reaction times were faster for targets on noise and slower on inverted images, compared to natural scenes and textures. The N2pc component of the event-related potential, a marker of attentional selection, had a shorter latency and a higher amplitude for targets in noise than for all other backgrounds. The background contralateral to the target had an effect similar to that on the target side: noise led to faster reactions and shorter N2pc latencies than natural scenes, although we observed no difference in N2pc amplitude. There were no interactions between the target side and the non-target side. Together, this shows that—at least when searching simple targets without own semantic content—natural scenes are more effective distractors than noise and that this results from higher-order statistics rather than from semantics or layout.

## Introduction

Traditionally, many studies on visual attention have used comparably simple stimuli such as geometrical shapes or letters. Although such research has been providing tremendous insight into the fundamental functions and mechanisms of attention, it also raises the question of how such findings transfer to more complex, naturalistic scenarios. As a first step toward the direction of higher ecological validity, over recent decades attentional guidance in natural scenes has become a topic of intense research. The term “natural” in this context refers to any scene one could encounter in real life, including indoors and manmade settings, and is not limited to outdoor scenes “in nature.” Transferring findings from simple to natural stimuli in general presents a non-trivial task. On an abstract level, the challenge can be attributed to nonlinearities in the processing chain: Responses to natural scenes cannot be described as a mere (linear) aggregation of responses to simple features and parts (Einhäuser & König, 2010). In turn, natural scenes are not an arbitrary agglomeration of pixels but instead obey specific statistical regularities. These regularities can occur on several levels, from low-level statistics through higher-order regularities (which may result in statistical rules reminiscent of Gestalt laws; Brunswik & Kamiya, 1953) to semantic content. This point raises a critical question for any neural or cognitive process: Up to which of those levels does a stimulus have to match a natural scene, such that it becomes indistinguishable from an actual natural scene for this particular process? In the present study, we investigated this issue for attentional guidance during visual search in natural scenes. Specifically, we asked (a) to what extent does a natural-scene background distract from target selection in visual search, if the search target itself is unrelated to the scene, and (b) which constituents of natural scenes (low-level statistics, higher-level statistics, semantics, or layout) are responsible for such distraction or interference?

One of the most widely used methods to assess attentional allocation in (natural) scene viewing is the measurement of gaze (for reviews, see Henderson, 2011; Schütz, Braun, & Gegenfurtner, 2011; Tatler, Hayhoe, Land, & Ballard, 2011). This approach rests on the assumption that gaze and attention are tightly linked under natural conditions, as supported by common neural substrates (e.g., Rizzolatti, Riggio, Dascola, & Umiltá, 1987) and by the observation that gaze shifts are typically preceded by shifts of attention to the subsequent fixation location (Deubel & Schneider, 1996). However, because gaze in scene viewing usually consists of an alternating sequence of fixations and saccades and because fixation durations themselves are influenced by a variety of factors (Nuthmann, 2017), there are limits to the temporal resolution when gaze is used as a proxy for attention. This is particularly critical immediately after image onset prior to the first saccade, where there is a predominance for the use of stimulus-driven information (Anderson, Ort, Kruijne, Meeter, & Donk, 2015), which in many situations is quickly overruled by task-related signals prior to the first saccade (Einhäuser, Rutishauser, & Koch, 2008). Hence, it is worthwhile to complement studies of gaze (i.e., of *overt* attention) with studies of *covert* attention (i.e., attentional allocation without gaze shifts) and to use methods of fine temporal resolution. In the present study, we therefore used electroencephalography (EEG) to assess the allocation of covert attention after the onset of a visual stimulus.

To study the availability of attentional resources on a more global scale, dual-task paradigms (Allport, Antonis, & Reynolds, 1972) are frequently used. In the context of natural scene viewing, these have demonstrated that processing the “gist” of a scene (e.g., deciding about the presence/absence of an animal or vehicle; cf. Thorpe, Fize, & Marlot, 1996) incurs little or no dual-task cost—neither when paired with a simple task (Li et al., 2002) nor when two gist processing tasks are conducted in parallel (Rousselet, Fabre-Thorpe, & Thorpe, 2002). This has been interpreted as gist processing occurring “in the (near) absence of attention” (Li, VanRullen, Koch, & Perona, 2002). This implies that the processing of the gist of a scene is rapid, automatic, and presumably pre-attentive. With gist processing of a scene happening pre-attentively, this raises the questions of whether and how the scene itself interferes with attentional processes that operate on the scene or with the scene as background (e.g., search for an item in the scene, either related or unrelated to the scene), and—if the scene does interfere with attentional processes—which constituents, properties, or features of the scene are responsible for this interference?

The ability to rapidly process natural scenes is also exploited and explored in so-called rapid serial visual presentation (RSVP) paradigms, where multiple objects or scenes are presented in rapid succession (Potter & Levy, 1969). In this case, attentional interference between subsequent scenes is observed (e.g., Einhäuser, Koch, & Makeig, 2007; Evans & Treisman, 2005). Like the attentional blink in simple items (Raymond, Shapiro, & Arnell, 1992), this interference is, however, more likely a consequence of limitations in later processing and memory access than of a limitation in early processing and feature analysis (Evans & Treisman, 2005; Yao & Einhäuser, 2008). This, in turn, again leads to the question of which constituents of a natural scene interfere with attentional processing.

Most studies on natural scene viewing use tasks that are related to the scene as such—for example, a present/absent judgment for a particular (broad) object category (such as an animal or a vehicle). Here, we followed a complementary approach by asking how *distracting* different constituents of a natural scene are when they are *not* task-relevant. To this end, we used a variant of visual search. Visual search is a paradigm not only widely studied with comparably simple stimuli (e.g., Treisman & Gelade, 1980) but also being increasingly applied to more natural scenes, more complex tasks, and more complex scenarios (for a recent review, see Wolfe, 2020). Plenty of this research has focused on properties of the search target and its relation to isolated distractors. Notable findings include that search difficulty depends on the similarity between actual target appearance and the target template (Vickery, King, & Jiang, 2005) and on the knowledge about the target (Schmidt & Zelinsky, 2009). Moreover (as for simple stimuli) a high similarity between target and distractors can impede search, even when object categories rather than templates are searched for (Alexander & Zelinsky, 2011; Alexander & Zelinsky, 2012). Here, we instead used a simple target, a Gabor patch that can occur at one of six locations, and thereby circumvented issues relating to target knowledge, target appearance, and the relation of the target to distracting items or backgrounds.

To study how a natural scene or its constituents interfere with (i.e., distract from) visual search, the Gabor patch targets in the present study were embedded in natural scenes or in modified versions of these scenes. This distinguishes our study from most studies on visual search in natural scenes, where observers search for natural objects occurring in the scene. In this case, the scene provides a context for attentional guidance, and target–scene consistency becomes critical. Such context effects were first described for overt attention during free viewing of line drawings, where out-of-context objects are fixated earlier, more frequently, and longer (Loftus & Mackworth, 1978). At least the longer fixation durations robustly transfer to visual search (Henderson, Weeks, & Hollingworth, 1999) and more photorealistic stimuli (Võ & Henderson, 2009). Similarly, consistency with the context facilitates search (Neider & Zelinsky, 2006), the probable target location given by the scene context can be used as prior knowledge to improve models of attention (Torralba, Oliva, Castelhano, & Henderson, 2006) and search often starts in regions likely to contain the target, even if it is in fact absent (Eckstein, Drescher, & Shimozaki, 2006). These, and many more studies along similar lines (for a review, see Võ & Wolfe, 2015), underline the importance of scene context for searching natural objects in natural scenes. The effects of “context” on attention also imply that it must be extracted rapidly and at least in part pre-attentively. This suggests that a “non-selective pathway” is involved in visual search in natural-scene backgrounds (Wolfe, Võ, Evans, & Greene, 2011). The “context” in visual search could in principle relate to gist or to the spatial layout of the scene or to more general statistical properties of the specific scene. In turn, these very scene properties could also interfere with target selection, as a complex background tends to slow search (Wolfe, Oliva, Horowitz, Butcher, & Bompas, 2002). Hence, when searching a natural object in a natural scene background, there are potentially two counteracting effects: interference of scene processing with search and contextual guidance. By using a Gabor patch as the target, fixed potential target locations, and scenes that have no extended horizontal surfaces, we avoided any contextual guidance based on spatial relations or physical constraints—that is, using the terminology of Võ and Henderson (2009), we prevented any “semantic” and “syntactic” relations of the target to the scene. By avoiding contextual guidance in our paradigm, we were able to isolate the *distracting* effects that natural scenes or their constituents exert on visual search.

The effect of background complexity on visual search has previously been assessed behaviorally. In a comprehensive study, Wolfe and colleagues (2002) measured reaction times in a present/absent judgment when varying set size and background complexity. They postulated three stages that are required to complete their search task: segmenting the item from the background (pre-attentive and non-selective), attentional selection per se, and item identification. Segmentation and identification could in principle be achieved for all items in parallel and would therefore be largely unaffected by set size. In contrast, the time required for attentional selection would scale with the number of items (i.e., there would be a positive search slope). In their first five experiments, Wolfe and colleagues (2002) found that increasing background complexity led to a general slowing (an additive, set-size-independent offset on search times), but left the search slope virtually unaffected. They concluded that in these cases background complexity primarily affected the segmentation and identification stages but had little effect on attentional selection. In their Experiment 6, however, the authors *did* find a clear effect of background complexity also on the search slope, suggesting that the background can indeed also affect the attentional selection stage. To assess which constituents of a natural-scene background interfere with attentional selection of an embedded target, we complemented behavioral (reaction time) measurements by an EEG measure that indexes the attentional selection—the so-called N2pc.

The N2pc is a negative deflection in the event-related potential (ERP) observed contralateral to an attended item (Luck & Hillyard, 1994). In early studies on the N2pc, this component was rather exclusively attributed to the attentional filtering of competing information (i.e., filtering out the distractor; Luck & Hillyard, 1994). More recent views give additional credit to target selection and enhanced target processing as contributors to the N2pc (e.g., Hickey, Di Lollo, & McDonald, 2009; for a recent review, see Stoletniy, Alekseeva, Babenko, Anokhina, & Yavna, 2022). By using the N2pc in addition to behavioral (reaction time) measures, we ensured that we were assessing attentional selection rather than non-selective processes such as target segmentation or identification.

The N2pc is often measured in search tasks in which two or more items compete for attentional priority. In a widely used variant, search items (target and distractors) are arranged equidistant from central fixation, observers are required to make a judgment related to the target (e.g., report the orientation of a line inside the target item), and one of the distractors (the “additional singleton”) is made salient by being distinct from the other items in a task-irrelevant dimension (Theeuwes, 1991; Theeuwes, 1992). The processing cost associated with the presence of the additional singleton has been interpreted as a result of attention being captured by the singleton—the most salient item in the display—before attention can be deployed to the target, an interpretation that has been supported by ERP markers of attentional selection such as the N2pc (Gaspelin & Luck, 2018; Theeuwes, 2010; but see Folk, Remington, & Johnston, 1992; Gaspelin, Leonard, & Luck, 2015; for an overview, see Luck, Gaspelin, Folk, Remington, & Theeuwes, 2021). Distraction from visual search, however, does not have to be limited to individual, isolated items but can result in principle from the whole visual stimulus at once. Likewise, the use of the N2pc in displays with multiple competing items is not limited to stimulus configurations as used in the additional singleton task. For example, Schubö, Wykowska, and Müller (2007) used stimulus arrays that consisted of line elements and formed either a homogeneous or a heterogeneous texture of vertically or horizontally oriented lines. They found a tendency for the N2pc to be smaller when targets and distractors were embedded in a random texture as compared to a background with more structure (i.e., fully or partially homogeneous backgrounds). The N2pc also tends to occur somewhat earlier for homogeneous than for heterogeneous background, although the bulk of behavioral speed-up of search for homogeneous backgrounds (cf. Duncan & Humphreys, 1989) is likely attributable to more efficient distractor suppression rather than to target selection (Feldmann-Wüstefeld & Schubö, 2013; Feldmann-Wüstefeld & Schubö, 2016). Here, we extend the approach of studying different background configurations to natural-scene backgrounds and to backgrounds that are matched to these natural scenes in different respects.

When addressing the question of which constituents of a natural-scene background interfere with target selection, we should note that the precise delimitations between distinct image-based factors vary considerably among research fields. In research on visual attention, often an arrangement roughly reflecting the processing hierarchy of the (ventral) visual system (Felleman & Van Essen, 1991; Riesenhuber & Poggio, 1999) is followed. Features are classified as low-level (e.g., luminance, contrast), mid-level (e.g., edges, corners, clutter), or high-level (e.g., proto-objects, objects). Lower-level features are often combined to compute the “salience” of a location (Itti & Koch, 2001) and then viewed as distinct from “semantics” (which includes factors relating to the meaning of a scene region; e.g., Henderson & Hayes, 2017) and “global scene structure” (Torralba, 2003). Among high-level features, objects play a special role, as objects guide attention (Egly, Driver, & Rafal, 1994; Stoll, Thrun, Nuthmann, & Einhäuser, 2015), but in turn attention may be a prerequisite for stable object representations (Rensink, 2000). Moreover, it is not clear to what extent object processing can be isolated from the meaning of the object (Rogers, Hodges, Lambon-Ralph, & Patterson, 2003).

In the field of scene statistics, there is also some ambiguity regarding where to draw the line between low-level and higher-level scene statistics. For the present purpose, we use “low-level” statistics synonymously with “up to second-order” statistics. That is, low-level refers to all the information that is contained in the autocorrelation function of a scene (or equivalently in its power spectrum, which is the Fourier transform of the autocorrelation, and the amplitude spectrum, which is the square root of the power spectrum). This usage is in line with studies on natural scene statistics (e.g., Field, 1987; van Hateren, 1992) and perception (Tadmor & Tolhurst, 1994). Higher-level statistics, in contrast, refers to scene structure not contained in the second-order statistics (cf. Franz & Schölkopf, 2004), and “semantic content” refers to identifiable, nameable items or objects. Semantics will include this “objective” content as well as the meaning an individual ascribes to it. The term “gist” of a scene refers to the content that can quickly be extracted from a scene (for a detailed discussion of the term, see Fei-Fei, Iyer, Koch, & Perona, 2007). Gist is distinct from scene layout, which can be perceived independent of objects, as demonstrated by hybrid images whose objects are in conflict with the scene layout (e.g., street scenes composed of furniture; Schyns & Oliva, 1994). It has been argued that the second-order statistics (in particular, the distribution of power across different orientations) suffices for gist extraction (Torralba & Oliva, 2003), although if this mechanism were dominant in human gist perception then observers should be more susceptible to image rotations than is actually observed (Guyonneau, Kirchner, & Thorpe, 2006).

Attention in visual search is not only guided by stimulus properties. In addition to the meaning that an individual ascribes to a scene, there are also task- and goal-related factors, such as the features of a search template (e.g., Geng & Wittkowski, 2019; see also Wolfe, 2021). Such factors are often subsumed as “top–down” to delimitate them from the stimulus-related “bottom–up” factors (e.g., Corbetta & Shulman, 2002; Desimone & Duncan, 1995). For example, additional singletons (Theeuwes, 1991) or abrupt onsets (Yantis & Jonides, 1984) capturing attention would typically fall in the bottom–up category, whereas attentional guidance driven by (known) properties of a target (Wolfe, Cave, & Franzel, 1989) would fall in the top–down category. The assumption of a bottom–up/top–down dichotomy has been challenged on conceptual grounds (Awh, Belopolsky, & Theeuwes, 2012) because, for example, the observer's prior selection history, especially with respect to previously reinforced stimuli, plays a decisive role in attentional guidance and does not fit in either the bottom–up or top–down category (Theeuwes, 2019).

In the context of natural scenes, additional challenges arise. Although some aspects are clearly bottom–up in the “stimulus-driven” meaning, such as salience defined by (local) feature contrasts (Itti & Koch, 2001; Nothdurft, 1992), and some aspects are clearly task-related (e.g., the target in search), the situation is less clear-cut for other aspects. For example, the gist or, more generally, the content of a scene in terms of object categories present and the setting (indoor/outdoor) are in principle independent of the observer and therefore stimulus-driven. On the other hand, at least part of the semantics can be open to interpretation by the observer and will also be modulated by their task and expertise (e.g., the same item could be identified as an animal, a dog, a specific breed of dog, a specific exemplar of dog), and individual differences have a profound influence on gaze-target selection in natural scenes (De Haas, Iakovidis, Schwarzkopf, & Gegenfurtner, 2019). Hence, in the present context, we divide semantics roughly into “content” (nameable things and stuff that are independent of the observer) and “meaning” (possibly observer-dependent interpretation of content). Consequently, categories such as bottom–up and top–down may not be easily applicable and might be replaced by more precise terms, such as “image-computable” (the information can be retrieved without any reference to a specific observer or context), “observer-dependent,” or “task-dependent.” In the context of the present study, we therefore focused on differential effects that specific parts of scene statistics have, which are all independent of the observer. In particular, the term “semantics” is used to reflect the content of a scene, which any observer could ascribe meaning to, without claiming that the interpretation ascribed is or is not specific to a particular observer. That is, we focus on factors related to the scene as such, rather than to their interpretation by the observer.

**Figure 1.**
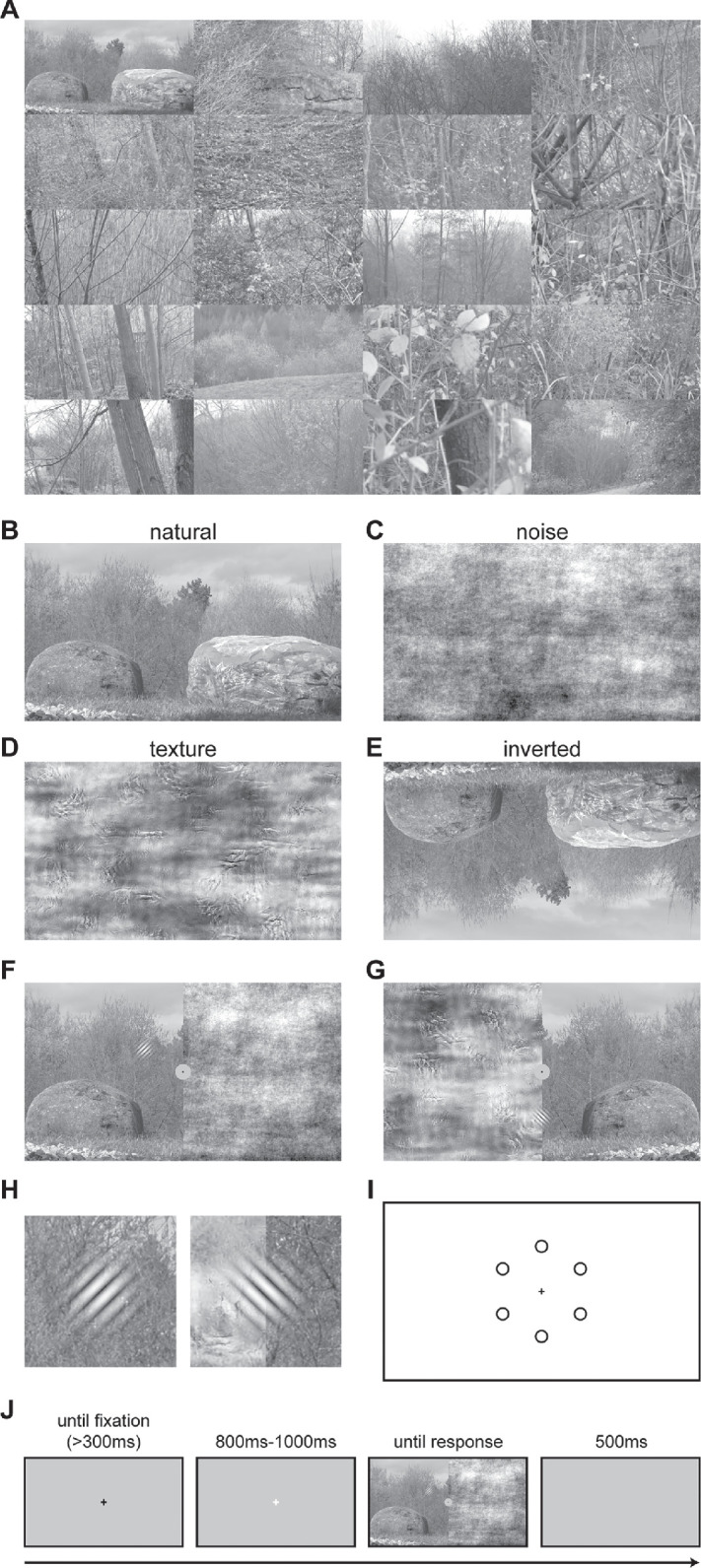
Stimuli and procedure. (**A**) Thumbnails of the 20 images used for all experiments; additional stimuli from the same database were used as fillers and for training. (**B**–**E**) Backgrounds: natural-scene background (used in all experiments) (**B**), noise background (used in Experiments 1 and 2) (**C**), texture background (used in Experiments 2 and 3) (**D**), and inverted (upside-down) background (used in Experiment 2) (**E**). (**F**, **G**) Example stimuli: split-scene stimulus (natural scene/noise) as used in Experiment 1 with right-tilted target in the upper left (**F**); split-scene stimulus (texture/natural scene) as used in Experiment 3 with left-tilted target on the vertical midline (**G**). (**H**) Zoom-in for targets in (**F**) and (**G**). (**I**) Potential target locations (fixation cross not to scale). (**J**) Schematic of a single trial (fixation crosses not to scale).

In sum, we differentiate between low-level statistics (up to second-order), higher-level statistics (beyond second-order), semantics (not differentiating the objective content from the meaning subjectively ascribed to it and including gist, as the rapidly extractable content), and scene layout (the basic geometric structure). To avoid confounds from subjective scene interpretation or expected target context, we used stimuli that depict outdoor scenes and are low in the content of nameable objects (Einhäuser & König, 2003) as background to a visual search task (Figures 1A–1D).

Across the three experiments, we used each scene in four different versions:

• First we used a grayscale image of the natural scene.
• Second, we used a noise stimulus created from the scene that matched the amplitude spectrum but was deprived of higher-order statistics (and thus also of semantic information and of layout) by randomizing the phase of the image. This exploits the fact that the amplitude spectrum of natural scenes follows a power function that falls off approximately with the inverse of the spatial frequency (1/*f*; Betsch, Einhäuser, Körding, & König, 2004; Ruderman & Bialek, 1994); that is, it has the spatial frequency characteristics of pink noise (1/*f* noise). Cardinal orientations usually carry more power than obliques (van der Schaaf & van Hateren, 1996) with the relation depending on scene category (Torralba & Oliva, 2003). Although humans can use amplitude information for slightly above-chance image classification (Wichmann, Braun, & Gegenfurtner, 2006), the bulk of perceived scene structure and content is carried by its phase (Piotrowski & Campbell, 1982). Hence, removing phase information from a scene provides a means to remove (nearly) all semantic content while keeping the low-level (second-order) statistical structure of the scene, including anisotropies in amplitude, intact.
• Third, we used a texturized version of each scene using the algorithm of Portilla and Simoncelli (2000) that preserves higher-order scene statistics while removing semantic content.
• Fourth, we used an upside-down (inverted) version of each scene. This inversion trivially changes the scene layout relative to the screen (unless the scene would be symmetrical at the horizontal midline, which natural scenes are not). Gist processing, in contrast, is relatively unimpaired by image rotation and thus by inversion (Guyonneau et al., 2006). However, there are some effects on later semantic processing; for example, unlike upright scenes, inverted scenes evoke no object–scene consistency effect, unless the object itself is also inverted (Lauer, Willenbockel, Maffongelli, & Võ, 2020). Inversion also may interfere with automatic processing of scene structure for contextual guidance, as evidenced in a change detection task (Kelley, Chun, & Chua, 2003). Nonetheless, in our present context, the major effect of inversion was on scene layout, and the effect of inversion on semantics (and its processing) was small compared to the other scene modifications.

The three experimental manipulations (noise, texture, inverted) therefore removed specific aspects from the original natural scene (Table 1): Inversion affects only or mostly scene layout, textures remove semantics and most of the layout but keep higher-level statistics, and pink noise removes layout, semantics, and higher-level statistics, keeping only low-level statistics.

**Table 1.** Background condition and corresponding image properties. Note that + indicates identical to natural scene; –, random or different from natural scene; (+), present but processing possibly impaired relative to natural scene; (–), absent but residual information might be available.

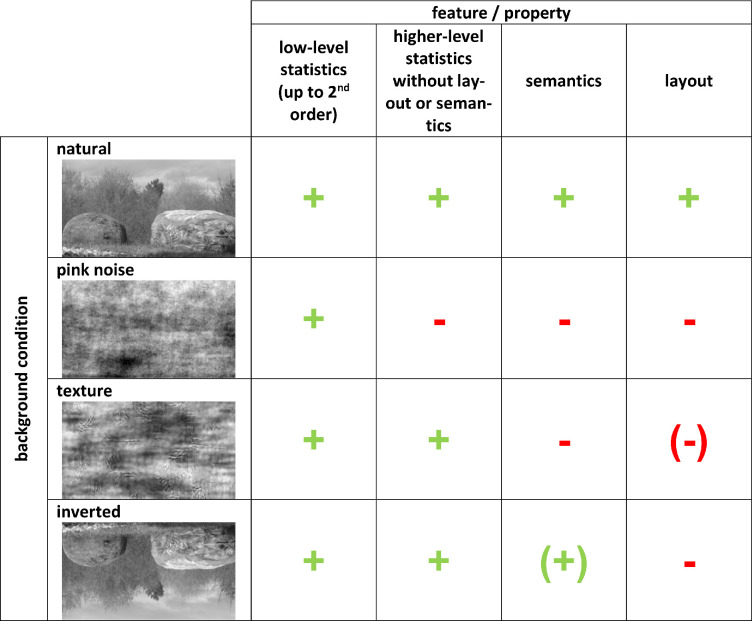	

Our approach to assessing whether natural-scene backgrounds interfere with the allocation of attention to the target and, if so, which constituents of the scene are responsible for such interference was motivated by the logic and design also employed in additional singleton tasks. Specifically, observers were asked to report the orientation of a target (a Gabor patch) that could occur at one of six locations equidistant to central fixation (on the left, on the right, or on the vertical midline). In this configuration, which is similar to many additional singleton and N2pc tasks (see above), the target can either be lateralized to one of the hemifields or be placed on the vertical midline. This allows us to quantify attentional allocation to the target by measuring the N2pc. The N2pc as an index of attention being allocated to the target is more specific as a marker of target selection than behavioral RT measures, which agglomerate a multitude of processes, both selective and non-selective. Rather than presenting additional non-targets and/or distractors simultaneously with the target, the target onset was accompanied by the simultaneous onset of the background scene. Hence, rather than measuring the effect of isolated distractors on attentional selection, we measure the distracting effect of the whole background.

We hypothesized that the four different backgrounds (noise, textures, inverted scene, and unmodified natural scene) would interfere with attentional selection of the target to various extents. A background of higher complexity (e.g., natural scene compared to noise) would then lead to stronger interference and would in consequence be associated with a longer reaction time (RT) and a delay in N2pc latency. Because each of the four different backgrounds matched the original natural scene in distinct respects (Table 1), we were able to determine which constituents of the scene (low-level statistics, higher-level statistics, semantics, and layout) are the main contributors to interference with target selection or (in more general terms) make search in a natural scene difficult. A background that causes less interference with target selection than unmodified natural scenes would cause shorter RTs and a decreased N2pc latency. In turn, the scene constituents that are lacking in this type of background (Table 1) are then implied to contribute significantly to such interference in the natural scene. If an RT effect was accompanied by a modulation of the N2pc, then we could more specifically attribute it to interference at the level of attentional selection.

The testing of our hypotheses was split into three distinct experiments. Experiment 1 investigated whether there are differences between natural scenes and noise. Finding a difference would show that low-level statistics alone do not yield the effect of the natural background on target selection. When using only backgrounds that are either natural or noise across the whole stimulus (“full scenes”), it would remain unclear whether interference arises from the relation of the target to its local surround (i.e., the salience of the target) or from global statistical properties of the background. To dissociate effects of local interference between target and the immediate surrounding background from those of global scene processing, we introduced an additional manipulation: some scenes were split at the vertical midline. Such split scenes had different backgrounds on either side, such that for lateralized targets the background of the target differed from the background on the other (non-target) side. In the split-scene conditions of Experiment 1, one side of the stimulus contained a natural scene and the other side noise. Effects of the non-target side on RTs and/or the N2pc would indicate that interference by the scene was not restricted to the local surround of the target.

In Experiment 2, we compared all four background conditions but excluded the split-scene condition to keep the overall experiment duration the same. Doing so allowed us to replicate Experiment 1 with respect to a noise/natural difference and also allowed distinguishing among higher-level statistics, semantics, and layout (Table 1). Finally, in Experiment 3, we directly compared texture stimuli and natural scenes both in a split scene and (partly replicating Experiment 2) in a full (non-split) configuration, again to ensure that the (dis-)similarity between the target and its surround did not drive or shadow potential effects. Note that split scenes between inverted and natural (upright) scenes were included in none of the experiments, as—by design of the stimuli and target locations (see Materials and Methods)—on average the surrounds of the targets in natural and inverted scenes were exactly identical. Hence, together the three experiments allowed us to pinpoint the main contributor (low-level statistics, higher-level statistics, semantics, or layout) to slowing search in natural-scene backgrounds and to determine whether these effects were local or acted throughout the visual field.

## Materials and methods

### Participants

A total of 60 healthy adult volunteers participated, evenly distributed across three experiments. In Experiment 1, 20 volunteers (two men, 18 women; 18 right-handed, two left-handed) between the ages of 19 and 32 years (average age, 23.5 ± 3.5 years) participated. In Experiment 2, 20 volunteers (nine men, 11 women; 16 right-handed, four left-handed) between the ages of 20 and 34 years (average age, 24.7 ± 4.0 years) participated. In Experiment 3, 20 volunteers (10 men, 10 women; 19 right-handed, one left-handed) between the ages of 20 and 33 years (average age, 24.5 ± 3.4 years) participated. The number of participants was planned prior to the study when applying for ethics approval, based on the comparable robustness of effects with small sample sizes in similar search studies (e.g., sample sizes between 10 and 12 per experiment [and group] in Wolfe et al., 2002; Schubö et al., 2007). Although no formal power analysis was performed, for the pairwise comparisons of interest a sample size of 20 would have corresponded to a power of 92% for alpha = 0.05 under the assumption of strong effects (*d_Z_* = 0.8). All 60 participants were naïve to the paradigm and hypotheses, had normal or corrected-to-normal vision, reported no history of neurological disorders, did not take any medication acting on the central nervous system, and gave written informed consent to their participation. All experimental procedures conformed to the tenets of the Declaration of Helsinki; the applicable body (Ethikkommission der Fakultät für Human-und Sozialwissenschaften, TU Chemnitz) determined that the experiments did not require in-depth ethics evaluation (case no. V121-WET-N2pc-22012016).

### Apparatus

Participants were seated comfortably in a chair in a sound-attenuated room with no source of light except for the screen. Participants were asked to use their right hand to press two buttons on the RESPONSEPixx Handheld response pad (VPixx Technologies, Saint-Bruno, QC, Canada). Stimuli were presented on a VIEWPixx /3D Lite LCD monitor (VPixx Technologies) with a resolution of 1920 × 1080 pixels and with a refresh rate of 120 Hz located 75 cm from the participant. Luminance ranged from 0.1 cd/m^2^ (“black”) to 94.4 cd/m^2^ (“white”). Images were presented on a medium gray background (47.8 cd/m^2^). In all experiments, eye movements were recorded with an EyeLink 1000 Plus device (SR Research, Ottawa, ON, Canada) in a desktop-mount configuration.

### Stimuli

#### Background

The main experimental manipulation concerned the complex background on which targets were presented. All experiments used stimuli based on 20 images from the Zurich Natural Image Database, which was originally co-created by one of the authors (Einhäuser et al., 2006; https://doi.org/10.5281/zenodo.10231379). The images depict outdoor scenes that contain no or very few man-made objects. Original resolution of the images was 2048 × 1536 in 24-bit RGB color. Images were converted to grayscale by the MATLAB rgb2gray.m function (MathWorks, Natick, MA) using default settings; the images were cropped to a resolution of 1920 × 1080 pixels (Figure 1A) to span the entire screen.

Across all experiments, four modifications of each of the 20 scenes were used:

• The unmodified stimulus is referred to as the “natural scene” throughout (Figure 1B).
• A stimulus for which higher-order information as well as semantic content were removed; to this end, we transferred the image to Fourier space, randomized the phases (preserving Hermitian symmetry), and transferred it back to image space. This resulted in an image that matched the respective natural stimulus in its amplitude spectrum but had a randomized phase spectrum. This stimulus is referred to as “noise” (Figure 1C). This method of phase randomization to remove higher-order statistics and preserve up to second-order statistics has been widely used (e.g., Einhäuser et al., 2006; Rainer, Lee, & Logothetis, 2004; Wichmann et al., 2006). For numerical reasons, some pixel values (between 0% and 0.8% of the image area) exceeded the displayable range; these were clipped to the maximum or minimum during presentation.
• A stimulus that was deprived of any semantic content but matched the natural stimulus with respect to higher-order properties; specifically, we used the algorithm proposed by Portilla and Simoncelli (2000) to generate these stimuli with an overcomplete complex wavelet transform. Parameters followed the default choices: four scales, four orientations, a spatial neighborhood size of 9 pixels, and 25 iterations. These stimuli are referred to as “textures” (Figure 1D). The fact that these textures mimic properties of natural scenes well is illustrated by the fact that they can become perceptually indistinguishable from a natural scene in the visual periphery under brief viewing conditions (e.g., Rosenholtz, 2011; Rosenholtz, Huang, & Ehinger, 2012).
• A stimulus that was identical to the natural stimulus but for which extraction of scene layout was more difficult; to this end, we used stimuli that were flipped upside-down, hereafter referred to as “inverted” scenes (Figure 1E). Such inversion trivially changes scene layout but leaves semantics largely intact (see Introduction for details).

These modifications were applied to all 20 stimuli in their original version and in a version that was flipped at the vertical midline (left–right) to balance any possible asymmetries. The resulting 40 (20 × 2) stimuli per modification were used as “background” in our experiments. Importantly, the effects of the modifications on contrast were negligible. We defined contrast as the standard deviation in an 80 × 80 patch divided by the image mean and averaging over the image (Einhäuser & König, 2003; Reinagel & Zador, 1999). This dimensionless contrast measure was slightly higher in the texture images and in the noise images than in the natural images (by 0.010 on average), but this difference was substantially below the standard deviation of contrast across all stimuli for any of the modifications (natural and inverted: 0.229 ± 0.065; noise: 0.238 ± 0.064; textures: 0.238 ± 0.062, where all values are expressed as mean ± *SD* across images).

For each experiment, additional stimuli of the same kind were created from different images of the same image database (Einhäuser et al., 2006) to be used in training and as fillers (see Procedure below).

#### Conditions

Throughout the experiments, we used two variants of the stimuli:

• Full scenes—Scenes that had the same modification throughout
• Split scenes—Scenes that differed in their modification in the left versus right hemifield

In either case, the stimulus to the left and the stimulus to the right of the vertical midline were modifications of the same original image.

The conditions differed among the experiments:

• In Experiment 1, we used natural scenes and noise as full scenes, as well as split scenes of natural scenes and noise (Figure 1F). This resulted in a total of four background conditions: full scene natural; full scene noise; split scene natural on the left, noise on the right; and split scene natural on the right and noise on the left.
• In Experiment 2, we aimed to address whether any differences observed between noise and natural scenes resulted from the difference in semantic content or from higher-order properties unrelated to semantics. To this end, we used textures and inverted-scene backgrounds in addition to noise and natural backgrounds but restricted the stimulus set to full scenes to keep the overall experimental duration identical.
• In Experiment 3, we tested whether a lack of difference between natural and texture backgrounds resulted from the exclusive use of full scenes, which unlike the split scenes do not induce any direct competition between the background types. Hence, we used full scenes and split scenes as in Experiment 1 but replaced noise by textures (Figure 1G).

#### Targets

The targets were Gabor wavelets (standard deviation (width) of the Gaussian envelope: 0.6°; spatial frequency of the grating: 1.9 cyc/°), which were tilted 30° either to the left or to the right (Figure 1H). In each trial, a target appeared simultaneously with the background at one of six distinct locations on the scene at 5.8° distance from fixation. Two of the potential target locations were in each hemifield, 4.9° left or right of the vertical midline and 2.9° above or below the horizontal midline. Targets at these locations are referred to as “lateral.” The two remaining target positions were on the vertical midline, 5.8° above or below a central fixation cross (Figure 1I). To embed the target seamlessly, it was blended in the background as follows. The Gabor was normalized such that its grating (*g*) oscillated between 0 and 1 and its Gaussian (*G*) was scaled to maximum 1 and minimum 0 (rather than to unit integral). The pixels of the grating were then pointwise weighted (multiplied) with the normalized envelope *G*, and the original image value (*I*) at the respective pixel was weighted (multiplied) by (1 – *G*). The resulting terms were added: *Gg* + (1 – *G*)*I* at each pixel. To simplify the task to maintain fixation during the presentation, a gray-filled circle (luminance: 47.8 cd/m^2^; radius: 1.0°), in which a black fixation cross was placed, was added in the middle of the screen (Figure 1G), referred to as “fixation area” hereafter.

### Procedure

The procedure was identical for all experiments (Figure 1J). After successful calibration and validation of the eye tracker, the block started. Each trial started with a fixation cross, which was shown until participants held fixation within a radius of 1.0° of the central spot for 300 ms; if they failed to do so within 3 seconds, the eye tracker was recalibrated. After correct fixation, the black fixation cross turned white. After an interval, whose duration was drawn from a uniform distribution between 800 ms and 1000 ms, the stimulus (consisting of background, target, and fixation area) was presented. Participants had to report the orientation of the Gabor target by button press while maintaining central fixation. The scene was replaced by a gray background 200 ms after the button press. The next trial commenced after 500 ms.

In each experiment, all 40 backgrounds (20 scenes and their left–right flipped versions) were used in all four conditions (depending on experiment; see “Conditions” section, above) and with each of the six target locations. This resulted in 960 (40 × 4 × 6) distinct stimuli, which were distributed across eight blocks of 120 trials each. The combinations of scene, background condition, and target location were distributed as uniformly as possible across these eight blocks. To this end, pairs of blocks (1 and 2, 3 and 4, 5 and 6, and 7 and 8) were treated as one entity. In each of these four block pairs, each of the 20 scenes was used 12 times and each combination of background and target location 10 times (five times per left/right version).

### Fixation control

Throughout the trial, eye position was monitored. Although all ERP paradigms typically require constant fixation, as eye movements introduce artifacts into the EEG signal, for lateralized ERP components, such as the N2pc, maintaining central fixation is particularly crucial, because, by definition, lateralization requires knowledge of the location of the (retinotopic) vertical meridian relative to the visual stimulus. In paradigms that use a circular arrangement of simple items (e.g., Grubert & Eimer, 2016; Hickey, McDonald, & Theeuwes, 2006; Kiss, Jolicœur, Dell'Acqua, & Eimer, 2008; Schubö, 2009; Woodman, Arita, & Luck, 2009), central fixation is usually beneficial for task performance and comparably easily achieved by participants. In these cases, fixation control can usually be deferred to offline analysis. In contrast, in our paradigm, participants can be inclined to move their eyes toward the target. Therefore, we introduced an online fixation control. Immediate feedback that a fixation had been broken (accompanied by a prolonged intertrial interval) incentivized participants to carefully follow the fixation instruction, even if they perceived it as impairing task performance. If the eye position fell outside a central circle of 1.5° radius (broken fixation), the scene disappeared immediately and a red fixation cross on a gray background was shown for 2 seconds. The next trial commenced after those 2 seconds. Trials in which fixations were broken were repeated at the end of the respective block, such that a block could eventually consist of more than 120 trials (yet, a block would always contain exactly those 120 trials with successfully held fixations containing the planned stimuli, thus ensuring counterbalancing for these trials, which were to be analyzed). To guarantee at least five trials between the original trial and its repetition, filler stimuli were inserted if needed. These filler stimuli used different images and were not analyzed. Relative to the first presentations (i.e., not considering fillers and trial repetitions) in each block, participants held fixation successfully in 90.7% (*SD* = 6.8%) of trials in Experiment 1, in 89.6% (*SD* = 10.6%) in Experiment 2, and in 92.7% (*SD* = 4.2%) of trials in Experiment 3. The fact that we still observed about 10% of broken fixation underlines the necessity to enforce central fixation. Detecting broken fixations immediately also allowed us to keep conditions and scenes counterbalanced, which was crucial as the analysis required the background on the target and the non-target side to be equivalent when averaged over all trials. Hence, *online* fixation control, which allows immediate feedback on broken fixations and repetition of trials with broken fixations, is particularly important for paradigms of this type.

### EEG recording

EEG was recorded with 58 active Ag/AgCl electrodes fixated on an electrode cap (ActiCap; Brain Products GmbH, Gilching, Germany). Additional electrodes to derive horizontal and vertical electrooculogram (EOG) were placed at the outer canthi of both eyes and below both eyes. Further electrodes were placed on the left and right mastoids, with the left mastoid serving as an online reference. The EEG signals were amplified and sampled at a 500-Hz rate using an actiCHamp EEG amplifier (Brain Products, Gilching, Germany).

### Data analysis

#### Behavioral data

The overall percentage of correct responses in each experiment was calculated after excluding filler trials and trials with broken fixations. To facilitate comparison with the EEG data, the condition-specific analysis of behavioral data included trials with lateral targets only (again excluding filler trials and trials with broken fixations). For these trials, we computed the percentage in which the direction of the Gabor patch was reported correctly in each background condition. For these correct trials, we computed the median RT for each participant. We considered the median to be the appropriate measure of the central tendency of RT distributions in each observer, as RTs typically do not follow a Gaussian distribution but rather a logarithmic Gaussian, rendering the arithmetic mean suboptimal. The geometric mean (or equivalently the arithmetic mean in log-space) might be considered as an alternative, but it is less robust than the median, which is why we consider the median preferable to characterize RTs per participant. Across participants these medians can be assumed to follow a normal distribution; therefore, means and parametric tests are considered appropriate. The effects of condition on percentage correct and on RTs were assessed by repeated-measures analyses of variance (rmANOVAs). For Experiments 1 and 3, we used the background on the side of the target and the background on the side opposite to the target as factors for a 2 × 2 rmANOVA. For Experiment 2, we used the background, which was identical on both sides (as only full scenes were used) as the factor in a 1 × 4 rmANOVA. Whenever sphericity was violated for the four-level factor (indicated by Mauchly's test at *p* < 0.05), a Greenhouse–Geisser correction was applied and the corrected *p* value is reported as *p*_GG_, along with the Greenhouse–Geisser ε and the original (uncorrected) degrees of freedom. Where appropriate, we conducted paired *t*-tests to follow up on significant effects in the rmANOVA. As a measure of effect size, generalized eta-squared (η*_G_*^2^) is reported in order to account for the repeated-measures design. According to Bakeman (2005), a value of η*_G_*^2^ = 0.02 can be considered a small effect size; η*_G_*^2^ = 0.13, a medium effect size; and η*_G_*^2^ = 0.26, a large effect size. In addition, partial η*_p_*^2^ is reported.

#### EEG data

EEG data were analyzed offline using MATLAB and EEGLAB 2022.1 (Delorme & Makeig, 2004). EEG data were re-referenced to the average of both mastoids, (M1 + M2)/2, high-pass filtered using a sinc finite impulse response (FIR) filter with a 0.1-Hz cutoff (Kaiser windowed, Kaiser β = 5.65, filter order 9056), and low-pass filtered using a sinc FIR filter with a 100-Hz cutoff (Kaiser windowed, Kaiser β = 5.65, filter order 184). Continuous EEG data were segmented into 500-ms epochs, including a 100-ms pre-stimulus baseline, with epochs time-locked to stimulus (target and background) onset.

An independent component analysis (ICA) was applied to attenuate eye-blink, lateral eye movement, and muscle artifacts (see Debener, Thorne, Schneider, & Viola, 2010). For ICA computations, a copy of the raw data was high-pass-filtered using a sinc FIR filter with a 1-Hz cutoff (Kaiser-windowed, Kaiser β = 5.65, filter order 906); epochs of 500-ms duration (–100 to 400 ms relative to stimulus onset) were generated, and epochs containing amplitude changes that exceeded 750 µV were removed (to avoid epochs with extreme artifacts deteriorating the ICA decomposition quality, whereas eye blinks and other remaining artefacts were separated by ICA into independent components for later removal). Independent components were computed using the extended infomax algorithm implemented in EEGLAB. ICA weights were transferred back to the pre-processed EEG data (i.e., with filter cutoffs 0.1 Hz and 100 Hz; see above).

Components identified as representing eye or muscle artifacts with a class probability of at least 80% (using ICLabel; Pion-Tonachini, Kreutz-Delgado, & Makeig, 2019) were removed from the data (on average, 8.4 components per participant were removed in Experiment 1, 8.2 components in Experiment 2, and 7.0 components in Experiment 3).

To extract activity associated with the processing of the lateral targets, we used electrodes over the left (PO3/PO7) and right (PO4/PO8) posterior–occipital regions in line with previous studies (Feldmann-Wüstefeld, Uengoer, & Schubö, 2015; Oemisch, Watson, Womelsdorf, & Schubö, 2017). We computed ERPs for electrodes contralateral and ipsilateral relative to the target in each of the four different background conditions. Difference waves were calculated by subtracting the activity recorded at electrodes ipsilateral to the lateral target position from the activity recorded at electrodes contralateral to the lateral target position.

In Experiments 1 and 3, ERPs and difference waveforms were calculated separately for the four background combinations with a 2 × 2 factorial design, with the background scene that was presented on the target side and the background scene that was presented on the side contralateral to the target (i.e., the non-target side). In Experiment 2, in which only full scenes were presented, ERPs and difference waveforms were calculated separately for the four types of background scenes (natural scene, inverted scene, texture, and noise).

##### Isolating target-related lateralization by using a midline target control

The so-far extracted contralateral-minus-ipsilateral difference waveforms contained the target-related N2pc activity but potentially also additional contributions from background processing. More specifically, split scenes that were used in Experiments 1 and 3 could result in background-specific lateralized processing; for example, when presenting a split-scene stimulus as depicted in Figure 1F, activity recorded at electrodes contralateral to the natural scene may differ from activity recorded at electrodes contralateral to noise due to a multitude of differences between the background types including local and global image features, as well as their capacity to capture attention. In order to isolate the target-related N2pc activity, we controlled for such background-specific effects. For each background condition, we used trials during which targets were presented on the midline as control. For these cases, differences between the hemifields cannot be caused by the target (as the target is on the midline). In an analogous manner to the lateralized target trials, we computed a “contralateral-minus-ipsilateral” waveform, with “contralateral” and “ipsilateral” now referring to the background type.

**Figure 2.**
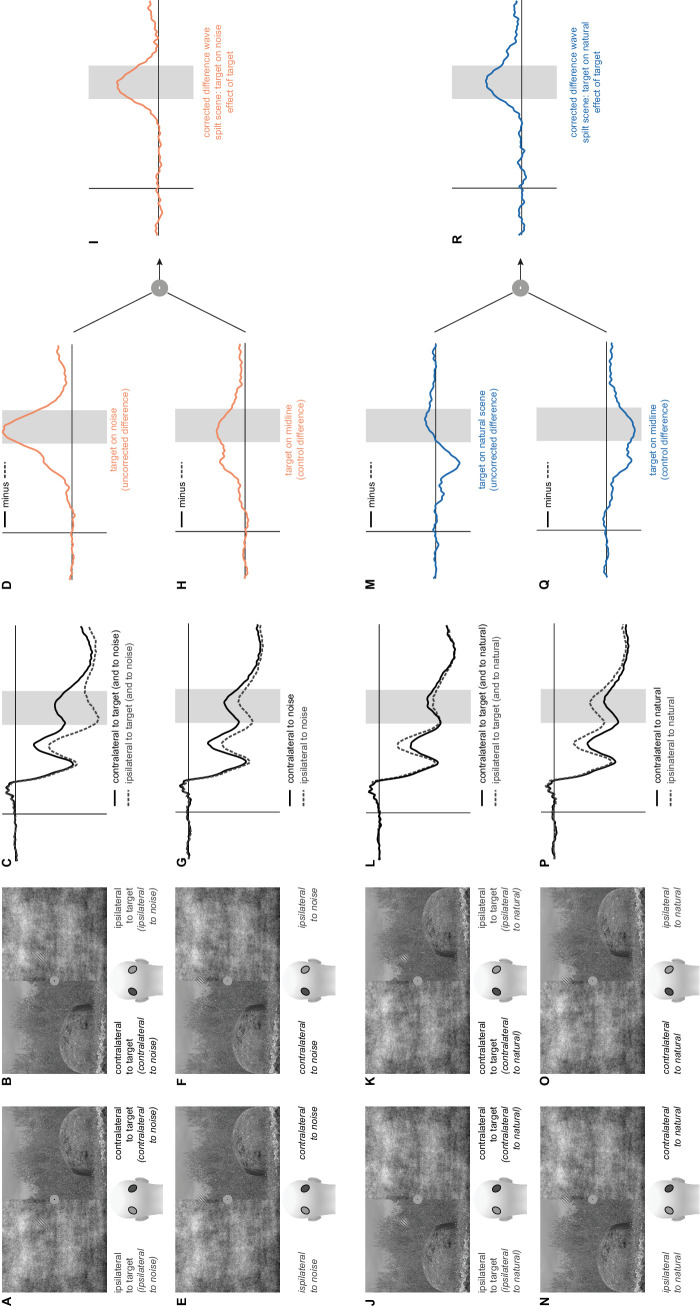
Illustration of the averaging and subtraction procedure to obtain corrected difference waveforms for split-scenes. (**A**, **B**) Examples of split scenes with the target in noise. (**C**) Average waveforms recorded ipsilateral (dashed) or contralateral (solid) to the target for the conditions of (**A**) and (**B**). (**D**) Difference waveform for (**C**). (**E**, **F**) Split scenes as in (**A**) and (**B**) but with targets on the midline; definitions of ipsilateral and contralateral sides are given by the background and are equivalent to (**A**) and (**B**). (**G**) Average waveforms for conditions of (**F**) and (**G**). (**H**) Difference waveform for (**G**). (**I**) Difference between waveforms of (**D**) and (**H**). (**J**, **K**) Examples of split scenes with the target on the natural scene. (**L**) Average waveforms recorded ipsilateral (dashed) or contralateral (solid) to the target for the conditions of (**J**) and (**K**). (**M**) Difference waveform for (**L**). (**N**, **O**) Split scenes as in (**J**) and (**K**) but with the target on the midline; definitions of ipsilateral and contralateral sides are given by the background and are equivalent to (**J**) and (**K**). (**P**) Average waveforms for conditions of (**N**) and (**O**). Note that ipsilateral and contralateral are flipped relative to (**G**), but otherwise the curves are mathematically identical. (**Q**) Difference waveform for (**P**). Note that the waveform is mathematically exactly the negative of (**H**). (**R**) Difference between waveforms of (**M**) and (**Q**).

We illustrate this procedure using two example conditions (Figure 2):

• First, we considered the condition with a split-scene background for which the target is embedded in noise (Figures 2A and 2B).
• To obtain the uncorrected difference waveform, we subtracted the ERPs recorded at the electrodes ipsilateral to the target from the electrodes contralateral to the target (Figures 2A–2C). Note that, for the condition under consideration, the subtraction always subtracted activity for electrodes ipsilateral to the noise (i.e., contralateral to the natural scene) from activity at electrodes contralateral to the noise. The resulting difference waveform (Figure 2D) therefore contained effects of target lateralization (i.e., N2pc-related activity) and of the difference between noise and natural scene.
• To isolate the effect of the target, we used stimuli with the target on the midline (Figures 2E, 2F) as control. We subtracted the ERPs at electrodes ipsilateral to the noise from the ERPs at electrodes contralateral to the noise (Figures 2E–2G). The resulting difference waveform (Figure 2H) contained only effects of the difference between the natural scene and noise.
• To obtain the corrected difference waveform containing only effects of the target (Figure 2I) (i.e., target-related N2pc activity), we subtracted the difference waveform obtained with targets on the midline (Figure 2H) from the difference waveform obtained with lateralized targets (Figure 2D). Note that, when applying the same logic to full scenes, the difference waveform obtained with targets on the midline (e.g., contralateral to noise minus ipsilateral to noise) is a zero line (as either hemisphere is contralateral as well as ipsilateral to noise).
• In the second, reverse example, where a split-scene background for which the target was embedded in the natural scene (Figures 2J and 2K), we applied the analogous procedure to obtain the uncorrected difference waveform (Figures 2L and 2M). Because the role of the background types was reversed with respect to their location relative to the target, the assignment of electrodes also had to be switched for the computation of the control (Figures 2N and 2O). Note that the resulting difference waveform (Figure 2Q) is mathematically exactly the negative of the difference waveform in the other condition (Figure 2H). Subtracting this waveform from the waveform of Figure 2M (or, equivalently, adding the waveform of Figure 2H) resulted in the corrected waveform for this condition (Figure 2R), which again contained only target-related effects.

This correction was only required for the split scenes, because for full scenes—thanks to using the original scenes and scenes flipped on the vertical midline (left/right) in a balanced fashion—the background was equivalent on the target and non-target sides.

To quantify the N2pc component, the most negative peak in an interval from 190 to 260 ms was identified in the individual corrected difference waveforms. To reduce the influence of ERP noise on the peak latency estimates, waveforms were Gaussian filtered (window length: 25 sampling points) prior to the extraction of peak latencies. In each of these Gaussian-filtered waveforms, we further quantified the time point at which 50% of the individual peak amplitude was reached, which is hereafter referred to as “N2pc slope latency” (for a similar procedure, see Berggren & Eimer, 2021; Liesefeld, 2018). N2pc amplitude was quantified by extracting the mean voltage from a 20-ms-wide window centered around the individual peak latency. Statistical analyses of latencies and amplitudes were performed analogously to the analysis of the behavioral data.

## Results

### Experiment 1

#### Behavior

In Experiment 1, we compared the detection of targets on a natural-scene background to the detection on a noise background, both in scenes that were uniformly natural or noise and in scenes that were split at the vertical midline between the two background versions. Because in split scenes the background was undefined for targets on the vertical midline, this analysis includes lateral targets only.

**Figure 3.**
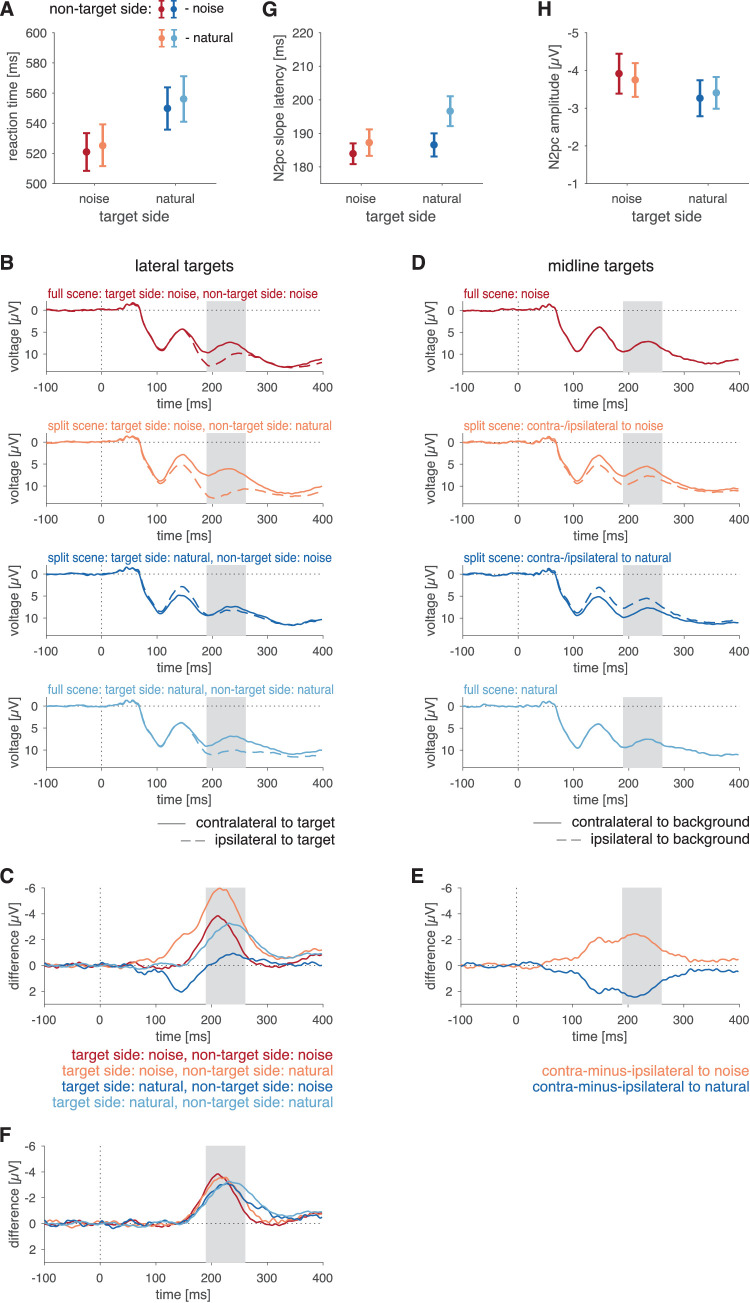
Results of Experiment 1. (**A**) Reaction time as a function of background combination. (**B**) ERPs at electrodes contralateral (solid curves) and ipsilateral (dashed curves) to the lateral targets in the four different background combinations. (**C**) Difference in signals between electrodes contralateral and ipsilateral to the targets for the four different background combinations for trials with lateral targets. (**D**) ERPs at electrodes contralateral (solid curves) and ipsilateral (dashed curves) to the background type for trials with the target on the vertical midline. Please note that, for full scenes, activity contralateral and ipsilateral to the background type is identical. For split scenes, the ERP at electrodes contralateral to the natural scene (solid dark blue curve) corresponds to the ERP at electrodes ipsilateral to noise (dashed orange curve) and vice versa. (**E**) Difference of split-scene conditions between signals at electrodes ipsilateral and contralateral to the noise side (orange curve) and to the natural-scene side (dark blue curve). (**F**) Curves of (**D**) corrected for the effect of scene lateralization in split scenes; that is, orange and dark blue curves correspond to those of (**C**) with curves of (**E**) subtracted; red and light blue curves are identical to the corresponding curves in (**C**). Shaded areas in (**B**) through (**F**) denote predefined N2pc intervals (190–260 ms). (**G**) N2pc slope latencies in each condition. (**H**) N2pc amplitudes in each condition. Error bars in (**A**), (**G**), and (**H**) denote standard error of the mean (*SEM*). Color codes for conditions are given in insets of the respective panels.

We found that participants performed the task correctly in the vast majority of trials (97.0% ± 4.1%). Also, we found no main effect of the background of the target on correctness, *F*(1, 19) = 3.09, *p* = 0.095, η*_G_*^2^ = 0.007, η*_p_*^2^ = 0.140, of the background on the other side, *F*(1, 19) = 0.39, *p* = 0.538, η*_G_*^2^ < 0.001, η*_p_*^2^ = 0.020, or an interaction between the factors, *F*(1, 19) = 1.12, *p* = 0.303, η*_G_*^2^ = 0.003, η*_p_*^2^ = 0.056. For correct trials only, we analyzed the median reaction time per participant and computed the arithmetic mean of these medians across observers (Figure 3A). The reaction time depended on the background of the target, *F*(1, 19) = 93.77, *p* < 0.001, η*_G_*^2^ = 0.057, η*_p_*^2^ = 0.832, with faster reactions for targets on noise than on natural scenes (see Table 2). Further, we found a main effect of the background on the side opposite to the target, *F*(1, 19) = 5.43, *p* = 0.031, η*_G_*^2^ = 0.002, η*_p_*^2^ = 0.222. When noise was presented on the side opposite to the target, reactions were faster than when the natural scene was presented on the opposite side. There was no interaction between the two factors, *F*(1, 19) = 0.16, *p* = 0.696, η*_G_*^2^ < 0.001, η*_p_*^2^ = 0.008.

**Table 2.** Mean values and standard deviation (SD) for correct response rate, reaction time, N2pc slope latency, and N2pc amplitude by background condition in each of the three experiments.

			Correct response rate, mean (*SD*)	Reaction time (ms), mean (*SD*)	N2pc slope latency (ms), mean (*SD*)	N2pc amplitude (µV), mean (*SD*)
Exp1	Target side	Non-target side				
	Natural	Natural	0.96 (0.05)	556.1 (67.4)	196.6 (19.9)	−3.41 (1.89)
	Natural	Noise	0.96 (0.06)	549.8 (62.6)	186.5 (15.4)	−3.26 (2.13)
	Noise	Natural	0.97 (0.06)	525.5 (61.8)	187.2 (17.6)	−3.75 (2.00)
	Noise	Noise	0.97 (0.04)	521.0 (56.0)	183.9 (13.8)	−3.92 (2.36)
Exp2	Background scene					
	Natural		0.97 (0.05)	568.2 (64.8)	196.8 (14.3)	−2.73 (1.26)
	Inverted		0.97 (0.05)	583.1 (66.9)	202.4 (21.0)	−2.79 (1.35)
	Texture		0.98 (0.04)	566.6 (63.9)	196.3 (20.4)	−2.61 (1.53)
	Noise		0.97 (0.04)	546.2 (63.5)	180.8 (13.4)	−3.42 (1.71)
Exp3	Target side	Non-target side				
	Natural	Natural	0.98 (0.02)	544.9 (65.6)	200.7 (20.4)	−2.78 (1.56)
	Natural	Texture	0.97 (0.03)	542.1 (73.0)	204.7 (26.3)	−2.63 (2.34)
	Texture	Natural	0.98 (0.02)	542.3 (71.8)	205.2 (22.8)	−2.28 (1.21)
	Texture	Texture	0.98 (0.02)	544.7 (67.4)	208.4 (20.9)	−2.36 (1.79)

#### EEG data

We computed ERPs contralateral and ipsilateral to the lateral target position for each of the four different background combinations (Figure 3B). Next, we subtracted the signal at electrodes ipsilateral to the target from the signal at electrodes contralateral to the target. The resulting contralateral-minus-ipsilateral difference waveforms (Figure 3C) show a pronounced negative peak in the predefined time window of interest for the N2pc (190–260 ms). However, in the split-scene conditions, these difference waves reflect a mixture of two effects: (a) the target-related N2pc and (b) a potential effect of the different backgrounds in the left and right hemifields. To estimate the lateralization effect caused by the background, we computed, in an analogous manner as for the lateral targets, ERPs for trials that had the target on the vertical midline (Figure 3D). The observed differences in the split-scene conditions (Figure 3D, difference between solid and dashed orange and dark blue curves) suggest that the side of the natural scene versus noise induces additional lateralization effects in the N2pc time window. To isolate effects of target processing lateralization (i.e., the target-related N2pc), we subtracted the difference waveforms obtained in split-scene trials with midline targets (Figure 3E) from the difference waveforms obtained in split-scene trials with lateral targets (Figure 3C, orange and dark blue curves) in each of the corresponding background conditions. The resulting differences (Figure 3F) reflect the effect of lateralized targets corrected for the effects induced by the scene split. These were used for quantitative analysis.

We retrieved slope latency and amplitude of the N2pc in the critical time window and tested how these depended on the background of the target (“target-side background”; two levels: noise and natural scene) and the background on the side opposite of the target (“non-target-side background”; two levels: noise and natural scene). For the N2pc slope latency (Figure 3G), we found a main effect of the target background, *F*(1, 19)  = 11.43, *p* = 0.003, η*_G_*^2^ = 0.032, η*_p_*^2^ = 0.376, with shorter latencies when the target was embedded in noise (Table 2). We further found a main effect of the background on the other, non-target side, *F*(1, 19) = 4.89, *p* = 0.039, η*_G_*^2^ = 0.040, η*_p_*^2^ = 0.205, with shorter latencies when noise was shown on the side opposite to the target. There was no interaction between the background on the target and the non-target side, *F*(1, 19) = 2.35, *p* = 0.142, η*_G_*^2^ = 0.011, η*_p_*^2^ = 0.110. That is, the N2pc latency mirrored the effects observed in the reaction-time data. For the N2pc amplitude, we found a main effect of the target background, *F*(1, 19) = 8.76, *p* = 0.008, η*_G_*^2^ = 0.015, η*_p_*^2^ = 0.316, with a lower amplitude when the target was on a natural-scene background than for the target embedded in noise (Figure 3F and Table 2). We observed neither a main effect of the non-target side, *F*(1, 19) = 0.01, *p* = 0.948, η*_G_*^2^ < 0.001, η*_p_*^2^ = 0.001, nor an interaction between the factors, *F*(1, 19) = 1.23, *p* = 0.281, η*_G_*^2^ = 0.001, η*_p_*^2^ = 0.061.

Experiment 1 demonstrated a dependence of behavioral and ERP measures on the target background. N2pc amplitudes were larger and both reaction times and N2pc latencies were shorter when the target was embedded in noise than when embedded in a natural scene. The background presented on the non-target side affected target processing as well, resulting in shorter reaction times and N2pc latencies for targets presented opposite to the noise (compared to the natural scene). Although the effects of the non-target side did not extend to the N2pc amplitude, they show that there was a contribution related not to the local surround of the target but to the processing of the scene as such. Our data demonstrate that a background containing only the low-level statistics of a natural scene does not interfere with target selection to the same extent as the natural scene does. Hence, other aspects of a natural scene, such as its higher-level statistics, semantics, or layout, must contribute to the interference of the background with target selection. Because pink noise (1/*f* noise) is deprived of all of these properties (Table 1), Experiment 1 alone could not distinguish between these alternatives. Hence, Experiment 2 used all of the modifications (noise, texture, and inverted) to disentangle these.

### Experiment 2

To dissociate the effects of higher-level scene statistics and semantics, in Experiment 2 we added conditions with a texture background. Textures are deprived of semantic content but preserve higher-level scene statistics (including some spatial, or layout, information). If semantics is responsible for the differences between natural scenes and noise observed in Experiment 1, this difference should persist if 1/*f* noise is replaced by textures. In turn, if higher-level statistics is responsible, the considered variables (RTs, N2pc latency, and amplitude) should be indistinguishable between natural scenes and textures and differ between each of these two backgrounds on the one hand and noise on the other hand.

A complementary explanation for the slowing of target processing in natural scenes relative to noise is based on the notion that scene layout is processed rapidly and in an automatic fashion. This notion is in line with the data of Schyns and Oliva (1994), who demonstrated that scene layout is processed independent from and extracted faster than objects in the scene (i.e., the scene content). Unless scenes are symmetric relative to the horizontal midline, inverting scenes trivially interferes with quick layout extraction. In the view of our approach, the presence of a natural-scene background acts akin to distractor stimuli in simple scenes, slowing down target processing by automatically capturing processing resources. Inverting scenes at the horizontal midline impairs the automatic extraction of scene layout. Hence, if automatically extracted scene layout plays a crucial role for slowing target selection in natural scenes, then inverted-scene (upside-down) backgrounds should be more similar to noise with respect to our dependent variables than upright natural-scene backgrounds. In turn, if scene layout is not the decisive factor that interferes with target selection in natural-scene backgrounds, our measures should be indistinguishable for inverted and upright backgrounds, as global statistical structure and semantic content are unaffected by the inversion. To keep the number of trials identical to Experiment 1 despite the two additional background conditions, only full scenes were used as backgrounds in Experiment 2.

#### Behavioral data

**Figure 4.**
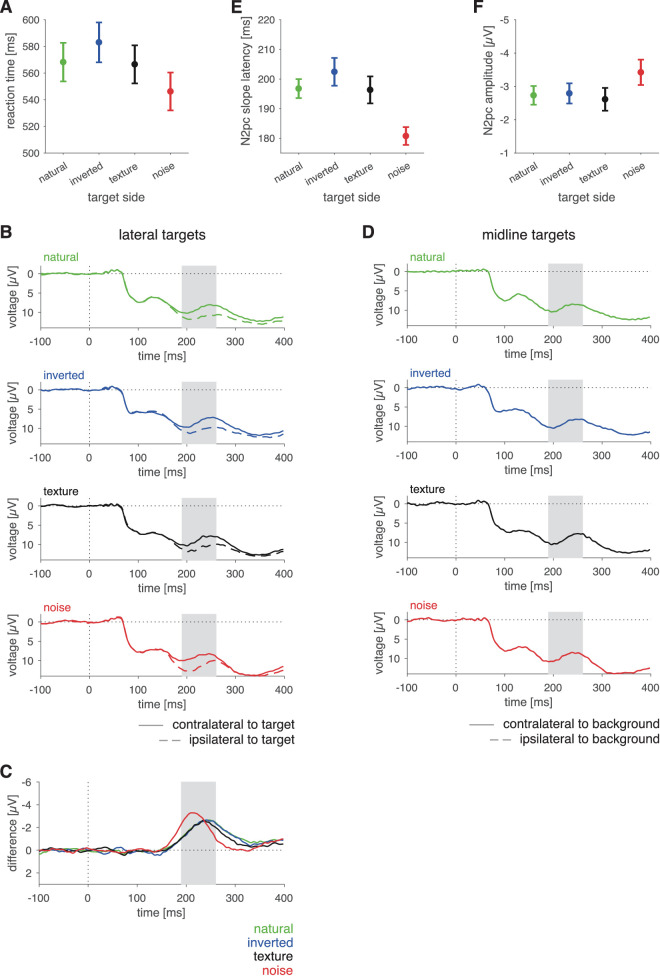
Results of Experiment 2. (**A**) Reaction time as a function of background condition. (**B**) ERPs at electrodes contralateral (solid curves) and ipsilateral (dashed curves) to the lateral targets in the four different background conditions. (**C**) Difference in signals between electrodes contralateral and ipsilateral to the targets for the four different background conditions for trials with lateral targets. (**D**) Average across the four electrodes of interest (PO3, PO4, PO7, PO8) for stimuli with targets on the midline, computed analogously to the split-scene controls in Experiment 1 (because only full scenes are used in Experiment 2, ipsilateral and contralateral backgrounds are identical for each electrode pair, thus the resulting ERP waveforms correspond to the average of PO3, PO4, PO7, and PO8). (**E**) N2pc slope latencies in each condition. (**F**) N2pc amplitudes in each condition. Error bars in (**A**), (**E**), and (**F**) denote *SEM*. Color codes for conditions are given in insets of the respective panels.

Participants performed the task correctly in 97.4% ± 4.1% of trials, with no dependence on the background, *F*(3, 57) = 3.04, *p*_GG_ = 0.073, ε = 0.530, η*_G_*^2^ = 0.008, η*_p_*^2^ = 0.138. We observed a significant main effect of background condition on reaction time, *F*(3, 57) = 29.46, *p* < 0.001, η*_G_*^2^ = 0.041, η*_p_*^2^ = 0.608 (Figure 4A). RTs were faster for targets on the noise background than for all other backgrounds, all *t*(19) > 5.46, all *p* < 0.001. RTs were slower for inverted scenes as compared to all other backgrounds, all *t*(19) > 3.44, *p* < 0.003, but we observed no significant differences in RTs between natural scene and texture backgrounds, *t*(19) = 0.52, *p* = 0.613.

#### EEG data

We first computed the ERPs at electrodes contralateral and ipsilateral to the lateral targets for each of the four background conditions (Figure 4B). Further, we subtracted the ERPs at electrodes ipsilateral to the target from the ERPs contralateral to target for each condition (Figure 4C). For the sake of completeness, we depict ERPs for trials with targets on the vertical midline for each of the four background conditions (Figure 4D); this corresponds to the computation of the correction for split scenes in Experiment 1 (Figure 3C).

In the predefined N2pc time window, we found a pronounced negativity for all background conditions. For the N2pc slope latency, we found a significant effect of background condition, *F*(3,57) = 15.78, *p* < 0.001, η*_G_*^2^ = 0.181, η*_p_*^2^ = 0.454 (Figure 4E). Follow-up tests confirmed that processing targets on noise was faster than on any other background; for noise versus natural, *t*(19) = 7.18, *p* < 0.001; for noise versus inverted, *t*(19) = 6.49, *p* < 0.001; for noise versus texture, *t*(19) = 4.76, *p* < 0.001 (Table 2), whereas no pairwise latency differences were found among natural scenes, inverted scenes, and textures, all *t*(19) < 1.86, all *p* > 0.08. The amplitude of the N2pc depended on the background condition, *F*(3, 57) = 8.80, *p* < 0.001, η*_G_*^2^ = 0.046, η*_p_*^2^ = 0.316 (Figure 4F). Follow-up tests showed that noise backgrounds differed significantly from natural scene, *t*(19) = 4.24, *p* < 0.001, inverted-scene, *t*(19) = 2.92, *p* = 0.009, and texture, *t*(19) = 5.13, *p* < 0.001 (Table 2) backgrounds, but no pairwise differences were found among natural scenes, inverted scenes, and textures, all *t*(19) < 0.97, all *p* > 0.345.

Together, the EEG data of Experiment 2 suggest that target processing on noise backgrounds differs from the other conditions, but differences among these conditions are, if anything, minute. Hence, the presence or absence of higher-level statistics rather than of semantic information seems to dominate early target processing on complex backgrounds, even though behavioral responses may be slowed down by the oddity of an inverted scene.

### Experiment 3

In Experiment 1, we found reaction times and N2pc latencies to be shorter when the background of the target contained noise as compared to natural-scene backgrounds. This also held for the background on the side opposite of the targets, independent of the background on the target side. That is, even remote from the target natural scenes acted as more effective distractors than noise. In Experiment 2, we found that textures were similar to natural scenes in these variables, and both textures and natural scenes were different from noise in reaction times, N2pc latency, and amplitude. This suggests that the differences observed in Experiment 1 arise from higher scene structure (present in textures and natural scenes but not in noise), but not from semantic content (present in natural scenes but not in textures nor in noise). However, Experiment 2 had no split-scene conditions; therefore, we could not distinguish between effects arising from processing the background as such or from impairment of target processing. Hence, in Experiment 3, we used split scenes consisting of natural scenes and textures along with full scenes of these backgrounds. If indeed textures are indistinguishable from natural scenes in terms of interfering with the allocation of attention to the target, unlike in Experiment 1, no effects of either side relative to the target should be observed on N2pc latency and reaction times. We used exactly the same design as in Experiment 1, except with noise replaced by textures.

#### Behavioral data

**Figure 5.**
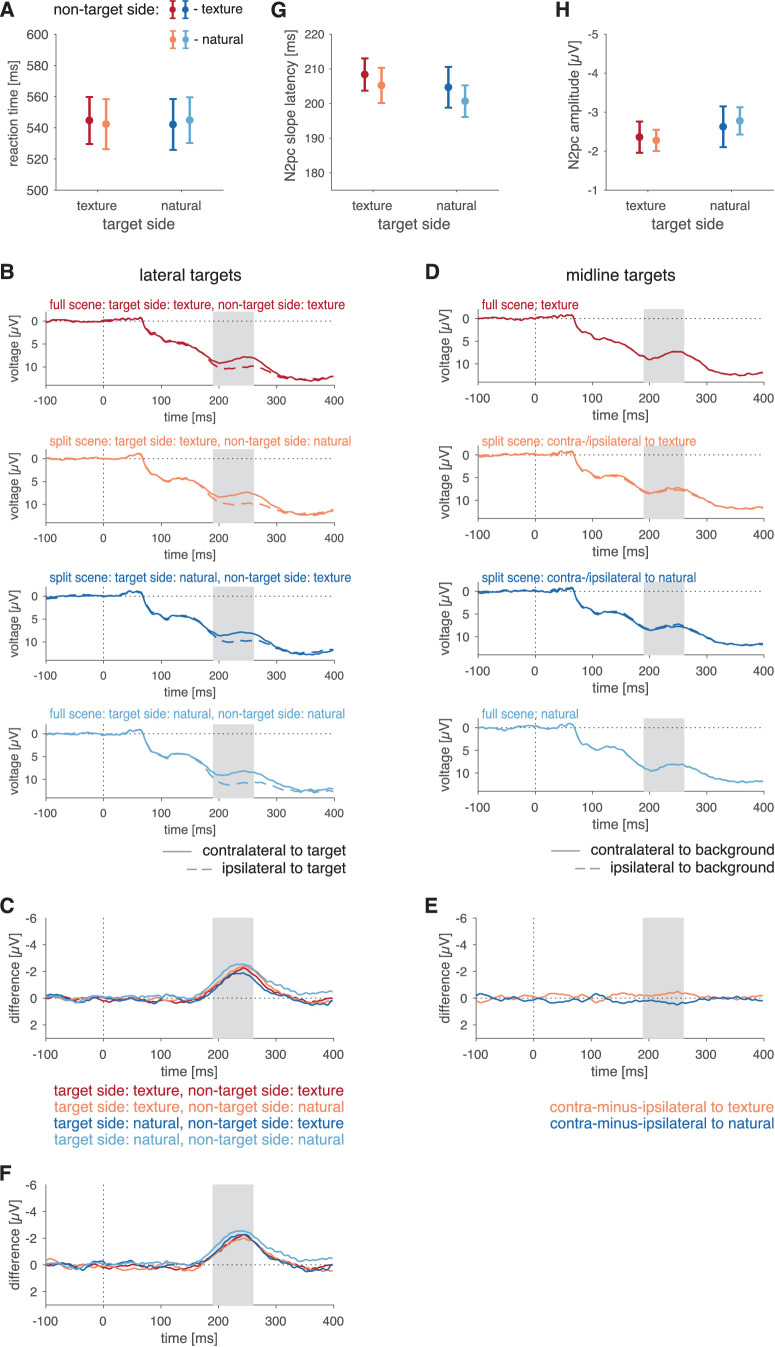
Results of Experiment 3. (**A**) Reaction time as a function of background combination. (**B**) ERPs at electrodes contralateral (solid curves) and ipsilateral (dashed curves) to the lateral targets in the four different background combinations. (**C**) Difference in signals between electrodes contralateral and ipsilateral to the target for the four different background combinations for trials with lateral targets. (**D**) ERPs at electrodes contralateral (solid curves) and ipsilateral (dashed curves) to the background type for trials with the target on the vertical midline. Please note that, for full scenes, activity contralateral and ipsilateral to the background type is identical. For split scenes, the ERP at electrodes contralateral to the natural scene (solid dark blue curve) corresponds to the ERP at electrodes ipsilateral to the texture (dashed orange curve) and vice versa. (**E**) Difference of split-scene conditions between signals at electrodes ipsilateral and contralateral to the texture side (orange curve) and to the natural-scene side (dark blue curve). (**F**) Curves of (**D**) corrected for the effect of scene lateralization in split scenes; that is, orange and dark blue curves correspond to those of (**C**) with curves of (**E**) subtracted; red and light blue curves are identical to the corresponding curves in (**C**). Shaded areas in (**B**) through (**F**) denote predefined N2pc intervals (190–260 ms). (**G**) N2pc slope latencies in each condition. (**H**) N2pc amplitudes in each condition. Error bars in (**A**), (**G**), and (**H**) denote *SEM*. Color codes for conditions are given in insets of the respective panels.

Participants performed the task correctly in 98.1% ± 1.5% of trials, and we found no significant main effect on correctness of the background on the target side, *F*(1, 19) = 1.85, *p* = 0.190, η*_G_*^2^ = 0.016, η*_p_*^2^ = 0.089, nor of the background on the other, non-target side, *F*(1, 19) = 0.11, *p* = 0.747, η*_G_*^2^ < 0.001, η*_p_*^2^ = 0.006, nor an interaction between these factors, *F*(1, 19) = 0.30, *p* = 0.589, η*_G_*^2^ = 0.002, η*_p_*^2^ = 0.016. For reaction times (Figure 5A), we found no main effects of the background on the target side, *F*(1, 19) < 0.01, *p* = 0.996, η*_G_*^2^ < 0.001, η*_p_*^2^ < 0.001, nor on the other side, *F*(1, 19) = 0.01, *p* = 0.926, η*_G_*^2^ < 0.001, η*_p_*^2^ < 0.001. There was no interaction between the two factors, *F*(1, 19) = 1.09, *p* = 0.309, η*_G_*^2^ < 0.001, η*_p_*^2^ = 0.054.

#### EEG data

Analysis of the EEG data followed the same procedure as in Experiment 1. First, trials with lateral targets were considered. ERPs were computed at electrodes contralateral and ipsilateral to the lateral targets for each of the four background combinations (Figure 5B). To obtain difference waveforms for each of the four background conditions, we averaged the electrodes contralateral to the target and subtracted the average of electrodes ipsilateral to the target from the resulting curves (Figure 5C). Next, ERPs for trials with targets on the vertical midline were computed (Figure 5D) corresponding to each of the background combinations of Figure 5B, respectively. To account for the effect of the scene split, we computed the contralateral-minus-ipsilateral-to-natural-scene ERP difference (Figure 5D, solid minus dashed dark blue curves) and contralateral-minus-ipsilateral-to-texture ERP difference (Figure 5D, solid minus dashed orange curves) for split scenes with targets on the vertical midline and subtracted the resulting curves (Figure 5E) from the difference waves for split scenes with lateral targets (Figure 5C). The resulting data (Figure 5F) were then used for quantitative analysis in the predefined N2pc time window. No effect of background was found on the N2pc slope latency (Figure 5G); for the main effect of target side, *F*(1, 19) = 1.33, *p* = 0.263, η*_G_*^2^ = 0.009, η*_p_*^2^ = 0.065; for the main effect of non-target side, *F*(1, 19) = 0.96, *p* = 0.339, η*_G_*^2^ = 0.006, η*_p_*^2^ = 0.048; and for the interaction, *F*(1, 19) = 0.01, *p* = 0.906, η*_G_*^2^ < 0.001, η*_p_*^2^ = 0.001. Similarly, for the N2pc amplitude (Figure 5H), we found little evidence for a main effect of the background on the target side, *F*(1, 19) = 4.13, *p* = 0.056, η*_G_*^2^ = 0.012, η*_p_*^2^ = 0.179. We found no effect of the non-target side, *F*(1, 19) = 0.02, *p* = 0.884, η*_G_*^2^ < 0.001, η*_p_*^2^ = 0.001, nor an interaction of the two factors, *F*(1, 19) = 2.19, *p* = 0.155, η*_G_*^2^ = 0.001, η*_p_*^2^ = 0.103. In sum, we found no ERP evidence for a difference in target processing between natural scenes and textures.

Consequently, Experiment 3 demonstrated that target selection is similar on natural scenes and on texture backgrounds and that this is true for the immediate surround of the target (no effects of target side), as well as for the global scene processing (no effects of non-target side). Taken together with the results of Experiments 1 and 2, this shows that there is a difference between noise, on the one hand, and textures, inverted scenes, and natural scenes, on the other hand. Because the difference between noise and the other background is the absence of higher-level statistics (Table 1), we have identified higher-level statistics (rather than semantics or layout) as the main contributor to the interference of a scene background with search.

## Discussion

In a series of three experiments, we compared the effects of complex backgrounds on target selection. In Experiment 1, we found that a noise background on the target side led to shorter N2pc latencies and faster reaction times, as well as higher N2pc amplitudes, than a natural-scene background. Interestingly, reaction time and target N2pc latency were also affected by the background opposite to the target (i.e., they were shorter when noise was presented on the opposite side compared to a natural scene background). This shows that the difference between natural-scene and noise backgrounds cannot be explained by the local target–background relation alone. Rather, the noise can be ignored more readily than the natural scene, either because the noise can be filtered out more quickly or because the natural scene interferes more effectively with allocating attention to the target. Put succinctly, natural scenes are more effective distractors than noise. In principle, this difference could arise from two factors: noise lacking higher-order scene statistics or noise lacking semantic content. To distinguish these alternatives, we used textures in Experiments 2 and 3 (Portilla & Simoncelli, 2000) that preserved higher-order structure but did not preserve semantic content. In all variables tested, natural-scene backgrounds were statistically indistinguishable from texture backgrounds (Experiments 2 and 3), although we observed a trend toward an effect of the target side on N2pc amplitude in Experiment 3. In contrast, texture backgrounds differed from noise backgrounds in the same way as natural-scene backgrounds did (Experiment 2). Together, these observations imply that higher-order scene structure distracts attention from the target (akin to salient distractors in simpler displays), irrespective of associated semantics or meaning.

In Experiment 2, we also tested inverted (upside-down) scenes as backgrounds. N2pc latency and amplitude were statistically indistinguishable between inverted scenes and (upright) natural scenes and textures. Inverted scenes showed similar differences as natural scenes and textures to the noise backgrounds. This suggests that it is not the scene layout that interferes with target selection in natural scenes compared to noise, but rather higher-order statistical regularities that are independent of scene inversion. Nonetheless, we found a behavioral slowing for inverted scenes (slower reaction times). The absence of similar N2pc latency effects, however, makes it unlikely that this slowing is mainly due to attentional allocation to the target. Instead, other (presumably later) stages of processing might interfere with the response for inverted scenes. The notion that later (post-perceptual) processes interfere with responses to natural scenes is in line with post-perceptual context effects of scenes in object detection (Hollingworth, 1998), which likely extends to inverted scenes (Lauer, Cornelissen, Draschkow, Willenbockel, & Võ, 2020).

The pattern of results we obtained with natural scenes and their modified variants is reminiscent of the pattern observed for background homogeneity in artificial stimuli. Homogeneous backgrounds yield quicker searches than heterogeneous backgrounds behaviorally (Duncan & Humphreys, 1989), as well as an earlier and somewhat larger target N2pc (Feldmann-Wüstefeld & Schubö, 2013; Feldmann-Wüstefeld & Schubö, 2016). We had hypothesized that we would confirm a slowing of search by natural scenes compared to at least one of the other backgrounds, isolating the property of the natural scene (low-level statistics, higher-level statistics, semantics, layout) that contributes to this slowing. Indeed, we found the bulk of the difference between 1/*f* noise, on the one hand, and all of the other backgrounds, on the other hand, suggesting that higher-level statistics are required for a background to slow search like a natural scene, but neither semantics nor layout (or at least semantics and layout have a much smaller effect on the observable measures than higher-level statistics as such).

The effect of background complexity on search has been addressed earlier with behavioral measures. In their six experiments, Wolfe and colleagues (2002) obtained slightly mixed results. Throughout their study, search slowed in general when the background became more complex. However, only when background and target were similar (their Experiment 6) did they find an effect on the search slope, which they interpreted as an effect of attentional selection (rather than a pre-attentive or a post-perceptual effect). With the N2pc we measured a component that is considered an index of the allocation of attention to a target (Stoletniy et al., 2022) and *did* find effects of the background. In the case of Wolfe et al. (2002), search items (potential targets and distractors) had to be individuated from the background, and search was then conducted among the set of these individuated (segmented) items. Hence, it is likely that pre-attentive segmentation, the mechanism to which they attributed the bulk of the slowing that did not affect the search slope, plays a more dominant role in their design. In our study, the actual search ended with finding the Gabor patch in the background, as there was always exactly one Gabor patch per trial. Hence, finding effects of background on attention allocation to the target as indexed by the N2pc in our study is consistent with Experiment 6 of Wolfe et al. (2002), which among their experiments had a design most closely related to ours. The comparison to Wolfe et al. (2002) also highlights the value of using the N2pc in addition to reaction times. Reaction times constitute an aggregate of many processes, including initial segmentation, attentional selection, target identification, and post-perceptual processes. Wolfe et al. (2002) used the search slope to isolate attentional selection, which required them to define a set size. In natural scenes, the set size is not readily definable (Wolfe, Alvarez, Rosenholtz, Kuzmova, & Sherman, 2011). For our research question, the background should not be disrupted by any search items other than the embedded target. Hence, rather than using multiple items, we complemented our behavioral measure by the N2pc latency and amplitude. This ensured that the differences we observed reflect the allocation of attention rather than non-selective segmentation or identification processes. Moreover, in the case of our scenes with little or no nameable content, searching for a single item (the target) among an otherwise unmodified background scene may be considered closer to an actual “natural” scenario than searching among a set of items placed artificially in the scene. Evidently, this may be different, if the search concerns a scene that is naturally rich in objects, such as a cluttered indoor room. In a similar vein, we deliberately refrained from using an actual object as target. Although this avoided any contextual effects arising from target-scene consistency and effects of target difficulty as such, it arguably limits the ecological validity of the search process. We consider this not to be critical, as oriented structures similar to the Gabor patch do occur “in the wild” including the background scenes, but considering a variety of targets in the present paradigm will be an interesting issue for further investigation.

Interestingly, when measuring ERPs in a detection task, effects of object–scene consistency can also be observed when scenes are replaced by meaningless backgrounds similar to our textures (Lauer et al., 2018). Lauer et al. (2018) attributed this result to “low-level” features. As these authors retained the color of their scenes (unlike in our stimuli), a contribution of color (a low-level feature) is highly likely, but it would be interesting to test how this contribution is modulated by the higher-level statistics retained in the textures used by Portilla and Simoncelli (2000).

Our experimental design deliberately prevented any contextual guidance. The target was unrelated to the scene, and the scenes themselves had no clearly discernable surfaces where real objects would be expected to occur. By using the same target throughout, we also avoided the learning of any scene–target association that could affect the N2pc in natural scenes (Patai, Doallo, & Nobre, 2012). Using the scenes merely as backgrounds rather than using natural targets also has the advantage of keeping the targets identical across conditions, reducing potential modulations by target features as such. This is complementary to a study that used the N2pc together with carefully photographed and selected natural stimuli to analyze the processing of natural targets in their typical context (Hickey, Pollicino, Bertazzoli, & Barbaro, 2019). In our study, we focused on the distracting effect of the scene as background and addressed how its features distract from unrelated targets. It might be an interesting further step to combine these two approaches—using stimuli that contain lateralized targets in their natural background and then independently manipulate target and background features. This would also shed further light on the relative contribution of scene properties as compared to contextual guidance in natural scene search.

In attention studies, 1/*f* noise stimuli have been used mostly as control or baseline stimuli against which natural-scene data are compared, making use of the fact that they preserve the low-level statistical structure of natural scenes without preserving their content. For example, effects of contrast on gaze decrease for 1/*f* stimuli as compared to their natural counterparts (Einhäuser et al., 2006), fixation durations increase (Einhäuser & Nuthmann, 2016; Kaspar & König, 2011) and saccade programming is modified (Walshe & Nuthmann, 2015). Our comparisons among natural scenes, textures (preserving higher-order structure, removing content) and noise (removing higher-order structure and content) suggest that the relevant differences for attentional selection are caused by higher-order statistics rather than by semantic content. In the light of the present data, it therefore seems advisable to use stimuli akin to the texture stimuli of Portilla and Simoncelli (2000) when only semantics are to be removed from natural scenes for control purposes.

Our results may also shed new light on the interpretation of the gist of a scene (Fei-Fei et al., 2007): If we follow the notion that gist and layout are those constituents of a natural scene that are extracted without requiring attention and that gist and layout are factors that drive attention (for example, to achieve object formation) (Rensink, 2000), only gist and layout could interfere with attentional processes related to target selection following scene onset. If gist processing were entirely based on low-level scene statistics (e.g., Torralba & Oliva, 2003), we would *not* be able to observe a difference between 1/*f* noise and any of the other conditions. The observation that gist goes beyond second-order statistics (Guyonneau et al., 2006) is therefore corroborated by the present findings. Conversely, not observing differences between textures and natural scenes could indicate that at least some information that relates to gist processing (i.e., is extracted pre-attentively) is conserved in these textures, in line with the aforementioned contextual cueing results (Lauer et al., 2018). Direct comparisons between the scene constituents that contribute to gist processing and those that interfere with subsequent target selection may be an interesting avenue for further research. In this context it should also be noted that our images are rather large compared to those used in most studies on semantics and gist (e.g., Li et al., 2002; Thorpe et al., 1996). Although one might assume that gist is extracted in parallel throughout the visual field (Rensink, 2000), in which case image size should be irrelevant, there is evidence for a spatial progression over time: Semantic processing typically evolves from the center to the periphery, but these spatiotemporal dynamics can be adjusted based on the typical spatial distribution of relevant information in the current experimental context (Larson, Freeman, Ringer, & Loschky, 2014). From rapid scene categorization, there is, in turn, evidence that peripheral vision might be *more* efficient than central vision for gist extraction (Trouilloud et al., 2020). It may therefore be of interest for both fields (gist processing and search) to assess the extent to which different parts of the visual field contribute differently to distraction from visual search. In the present study, however, we were interested in the effect of the scene as a whole, which is why we considered large scenes appropriate and which is helped by the fact that our scenes had little content that would be bound to a specific spatial scale.

On a neural level, responses in early visual areas as measured by functional magnetic resonance imaging (fMRI) tend to be indistinguishable between 1/*f* noise stimuli and their actual natural counterparts, whereas removing amplitude information (by “whitening” or using white noise) reduces early visual responses (Olman, Ugurbil, Schrater, & Kersten, 2004). This indicates that at an early level, processing of 1/*f* noise stimuli and natural scenes relies on similar mechanisms. The difference we observe between natural scenes and noise therefore likely occurs in later stages of processing. This is consistent with the N2pc as a marker of attentional selection (Eimer, 2014) and with the N2pc originating later and at more anterior sites than signals related to feature processing (Hopf, Boelmans, Schoenfeld, Luck, & Heinze, 2004). Movshon and Simoncelli (2014) reported a direct comparison among neural responses to noise, texture, and natural-scene stimuli akin to the stimuli used here for several areas of the ventral stream. Based on non-human primate data for V1 and V2 and human fMRI data for large parts of the ventral stream, textures, 1/*f* noise and natural scenes are indistinguishable in V1; textures and natural scenes alike yield different responses from 1/*f* noise in V2 and all extrastriate ventral areas; and differences between textures and natural scenes become only evident downstream to V3 (i.e., in the more anterior part of the ventral stream). Although methods and paradigms in these studies are vastly different from our visual-search approach, together they suggest the following interpretation: To effectively interfere with attentional selection, a background has to be processed prior to the allocation of attention to the target. In early vision, only 1/*f* noise distinguishes itself from the other stimuli tested, whereas textures and natural scenes are largely processed equally. Textures and natural scenes become distinguishable only at the stage of the ventral pathway where feedforward information and feedback information are first combined, V4 (Mazer & Gallant, 2003). It is notable that the dividing line we observe for interference with target selection after scene onset matches the dividing line observed for representations in areas earlier than V4 in the visual hierarchy.

In conclusion, we found that backgrounds containing higher-order scene statistics (natural scenes, textures, inverted scenes) interfered more strongly with target selection than backgrounds deprived of higher-order structure (1/*f* noise). In contrast, this interference was similar between natural-scene backgrounds and backgrounds deprived only of semantic content (textures) or layout (inverted scenes). Hence, the impact of natural-scene processing on attentional selection depends on higher-order scene statistics but not necessarily on semantic content or scene layout.
